# Popular Fallacies

**Published:** 1880-06

**Authors:** 


### POPULAR FALLACIES.

NIGHT air and damp weather are held in great horror by multitudes of persons who are sickly or of weak constitutions; consequently, by avoiding the night air, and damp weather, and changeable weather, and weather that is considered too hot or too cold, they are kept within doors the much largest portion of their time, and as a matter of course continue invalids, more and more ripening for the grave every hour; the reason is, they are breathing an impure atmosphere nineteen-twentieths of their whole existence.

As nothing can wash us clean but pure water, so nothing can cleanse the blood, nothing can make health-giving blood, but the agency of pure air. So great is the tendency of the blood to become impure in consequence of waste and useless matters mixing with it as it passes through the body, that it requires a hogshead of air every hour of our lives to unload it of these impurities; but in proportion as this air is vitiated, in such proportion does it infallibly fail to relieve the blood of these impurities, and impure blood is the foundation of all disease. The great fact that those who are out of doors most, summer and winter, day and night, rain or shine, have the best health the world over, does of itself falsify the general impression that night air or any other out-door air is unhealthy as compared with in-door air at the same time.

Air is the great necessity of life; so much so, that if deprived of it for a moment, we perish; and so constant is the necessity of the blood for contact with the atmosphere, that every drop in the body is exposed to the air through the medium of the lungs every two minutes and a half of our existence.

Whatever may be the impurity of the out-door air of any locality, the in-door air of that locality is still more impure, because of the dust, and decaying and odoriferous matters which are found in all dwellings. Besides, how can in-door air be more healthy than the out-door air, other things being equal, when the dwelling is supplied with air from without?

To this very general law, there is one exception, which it is of the highest importance to note. When the days are hot, and the nights cool, there are periods of time within each twenty-four hours, when it is safest to be in doors, with doors and windows closed; that is to say, for the hour or two including sunrise and sunset, because about sunset the air cools, and the vapors which the heats of the day have caused to ascend far above us, condense and settle near the surface of the earth, so as to be breathed by the inhabitants; as the night grows colder, these vapors sink lower, and are within a foot or two of the earth, so they are not breathed. As the sun rises, these same vapors are warmed, and begin to ascend, to be breathed again, but as the air becomes warmer, they are carried so far above our heads as to be innocuous. Thus it is that the old citizens of Charleston, South Carolina, remember, that while it was considered important to live in the country during the summer, the common observation of the people originated the custom of riding into town, not in the cool of the evening or of the morning, but in the middle of the day. They did not understand the philosophy, but they observed the fact that those who came to the city at mid-day remained well, while those who did so early or late suffered from it.

All strangers at Rome are cautioned not to cross the Pontine marshes *after* the heat of the day is over. Sixteen of a ship's crew touching at one of the West India islands slept on shore several nights, and thirteen of them died of yellow fever in a few days, while of two hundred and eighty who were freely ashore during the day, not a single case of illness occurred. The marshes above named are crossed in six or eight hours, and many travellers who do it in the night are attacked with mortal fevers. This does, at first sight, seem to indicate that night air *is* unwholesome, at least in the locality of virulent malarias, but there is no direct proof that the air about sunrise and sunset is not that which is productive of the mischief.

For the sake of eliciting the observations of intelligent men, we present our Theory on this subject.

A person might cross these marshes with impunity, who would set out on his journey an hour or two after sundown, and finish it an hour or two before sun-up, especially if he began that journey on a hearty meal, because, in this way, he would be travelling in the cool of the night, which coolness keeps the malaria so near the surface of the earth as to prevent its being breathed to a hurtful extent.

But if it is deadly to sleep out of doors all night in a malarial locality, would it be necessarily fatal to sleep in a house in such a locality? It would not. It would be safer to sleep in the house, especially if the windows and doors were closed. The reason is, that the house has been warmed during the day, and if kept closed, it remains much warmer during the night in-doors, than it is out-doors, consequently the malaria is kept by this warmth so high above the head, and so rarified, as to be comparatively harmless. This may seem to some too nice a distinction altogether, but it will be found throughout the world of nature, that the works of the Almighty are most strikingly beautiful in their minutiæ, and these minutiæ are the foundation of his mightiest manifestations.

Thus it is, too, that what we call "Fever and Ague," might be banished from the country as a general disease, if two things were done.

1. Have a fire kindled every morning at daylight, from spring to fall, in the family room, to which all the family should repair from their chambers, and there remain until breakfast is taken.

2. Let a fire be kindled in the family room a short time before sundown; let every member of the family repair to it, and there remain until supper is taken.

In both cases, the philosophy of the course marked out consists in two things.

First. The fire rarifies the malaria and causes it to ascend above the breathing point.

Second. The food taken into the stomach creates an activity of circulation which repels disease.

